# Rate of oophorectomy in pediatric ovarian torsion: risk factors and change over time

**DOI:** 10.1007/s00383-024-05743-8

**Published:** 2024-06-24

**Authors:** Joy Ayemoba, Kylie Callier, Kevin Johnson

**Affiliations:** 1 Department of Surgery, Division of Pediatric Surgery, The University of Chicago Medicine, Chicago, IL USA; 2 Department of Pediatric Surgery, Monroe Carell Jr. Children’s Hospital at Vanderbilt, Doctor’s Office Tower, Vanderbilt University School of Medicine, 2200 Children’s Way, Suite 7100, Nashville, TN USA

**Keywords:** Ovarian torsion, Oophorectomy, Outcomes

## Abstract

**Purpose:**

The management of ovarian torsion in pediatric patients has evolved over time. Ovarian salvage is currently recommended given concerns for fertility preservation and the low likelihood of malignancy. Studies have shown that the incidence of oophorectomy is higher amongst pediatric surgeons in comparison to gynecologists. Using a national database, this study examined how the surgical management of ovarian torsion has evolved.

**Methods:**

Children with a discharge diagnosis of ovarian torsion (ICD-9 code 620.5, ICD-10 code N835X) and procedure codes for oophorectomy (CCS code 119) were identified within the KID database from 2003, 2006, 2009, 2012, 2016, and 2019. Diagnosis of ovarian pathology was based upon ICD-9 and ICD-10 codes at the time of discharge.

**Results:**

A total of 7008 patients, ages 1–20, had a discharge diagnosis of ovarian torsion. Of those patients, 2,597 (37.1%) were diagnosed with an ovarian cyst, 1560 (22.2%) were diagnosed with a benign ovarian neoplasm, and 30 (0.4%) were diagnosed with a malignant neoplasm. There was a decreased risk of oophorectomy in urban-teaching versus rural hospitals (OR: 0.64, *p* < 0.001). The rate of oophorectomy has decreased overtime. However, patients with benign or malignant neoplasms were more likely to undergo oophorectomy than those without a diagnosis (OR: 2.03, *p* < 0.001; 4.82, *p* < 0.001).

**Conclusion:**

The rate of oophorectomy amongst children with ovarian torsion has decreased over time. Yet, despite improvements, oophorectomy is common amongst patients with benign ovarian neoplasms and those treated at rural hospitals. Continued education is needed to optimize patient care in all clinical scenarios.

**Level of evidence:**

IV.

## Introduction

Ovarian torsion is rare in the pediatric population. Analysis of the Kids’ Inpatient Database (KID) in 2006 estimated an incidence of 4.9:100,000 [1]. Although uncommon, it must be included in the differential diagnosis of any female presenting with abdominal pain as management involves urgent surgical intervention. Options for treatment include detorsion (with or without oophoropexy), cystectomy, and oophorectomy [2–4]. Traditionally, resection of the affected ovary has been the treatment of choice. However, over time there has been a shift in practice recommendations towards ovarian preservation.

Current literature strongly recommends conservative management with detorsion [5–7]. This approach has been shown to be effective and is thought to maximize the future reproductive potential of the patient [8]. However, despite increasing evidence of the benefits and safety of ovarian sparing techniques, the majority of patients continue to undergo resection. Concerns about an underlying malignancy, causing an embolic event, and/or the consequences of leaving unviable, necrotic tissue behind have led to the persistent high rates of oophorectomy [3, 9]. The rationale behind this approach was to remove a possible malignancy as well as avoid the risk of thromboembolism secondary to a thrombosed ovarian vein. In addition, delays in diagnosis and treatment were thought to limit the viability of the ovary. However, the risk of malignancy is quite low in this age group. A review of the literature by McGovern et al, found only two cases of pulmonary embolism associated with ovarian torsion (N = 981), both of which the patients underwent oophorectomy [4]. There has been no report of thromboembolism associated with ovarian torsion in the literature to date [3].

Despite existing literature, oophorectomy remains a common practice when faced with pediatric ovarian torsion [10]. Moreover, a study by Campbell et al. found that pediatric surgeons were more likely than gynecologists to perform oophorectomy in cases of ovarian torsion [11].

Therefore, a closer examination of evolving clinical practice is warranted. For the purposes of this current study, we sought to evaluate how the surgical management of ovarian torsion has evolved over time. Utilizing a national database, we aimed to better understand how practices have changed amongst pediatric surgeons in order to identify any areas for improved management of this condition.

## Methods

Our study was a retrospective cohort study utilizing data collected from the Kids’ Inpatient Database (KID). This study was deemed exempt from review by the corresponding Institutional Review Board (IRB). Following determination of exemption status, data was reviewed for the following years: 2003, 2006, 2009, 2012, 2016, and 2019.

Patients aged 1–20 years old with a discharge diagnosis of ovarian torsion (ICD-9 code 620.5, ICD-10 code N835X) were included within this study. The primary outcome of interest was oophorectomy in confirmed cases of ovarian torsion. Procedure codes for oophorectomy (CCS code 119) were included. All ICD and CCS codes were based upon data provided at time of discharge; therefore, combating the potential for patients with incorrect pre-operative diagnoses within the study cohort. This study excluded patients younger than 1-years-old to avoid including prenatal ovarian pathology and/or torsion.

Notable risk factors explored within this study were: year, hospital type, income, and diagnosis of ovarian pathology. As aforementioned, diagnosis of ovarian pathology was based on the ICD-9 and ICD-10 codes at the time of discharge.

A descriptive analysis was conducted using Stata 18.0 SE (College Station, TX) to explore the rates of oophorectomy in relation to selected risk factors. Data was scrubbed to avoid duplicate entries. Patients with multiple reported pathologies at the time of discharge were classified under the most clinically significant category (malignant neoplasm > benign neoplasm > ovarian cyst). Following a descriptive analysis, a univariate logistic regression model was created exploring each risk factor with the likelihood of oophorectomy. All statistically significant factors were included within a multivariate logistic regression model.

## Results

**Fig. 1:**
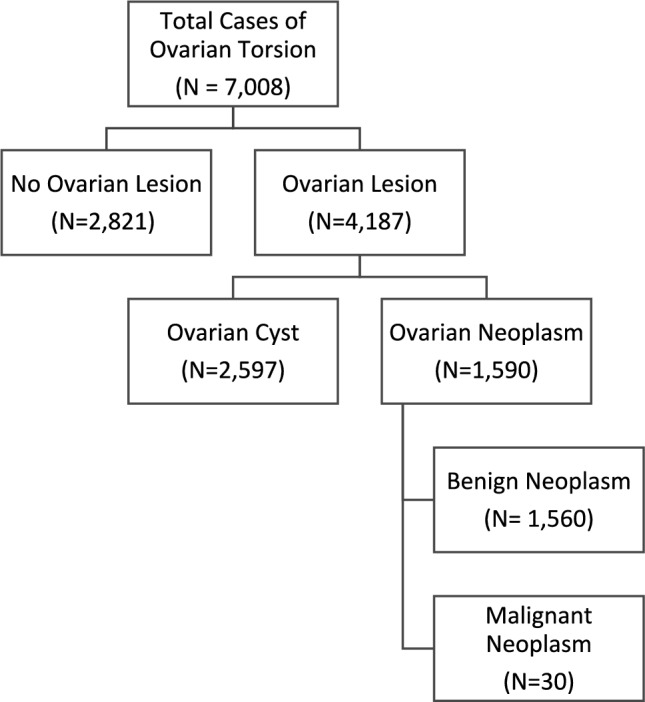
Overview of study population

Within the KID database, there were 7008 patients who were diagnosed with ovarian torsion. Amongst these patients, 2821 individuals (40.25%) had no formal diagnosis of an ovarian lesion at the time of discharge. Of those with diagnoses, 30 patients were identified as having a malignant neoplasm; 1,560 with a benign neoplasm, and 2597 with an ovarian cyst (Fig. 1). Within this cohort, an oophorectomy was performed in 3743 patients (53.41%) (Table 1). Almost half of all children with ovarian torsion with no identifiable pathology underwent oophorectomy (*n* = 1,386, 49.13%). Rates of oophorectomy were similar in children with ovarian cyst (50.6%). However, in children diagnosed with neoplasm there were significantly higher rates of oophorectomy performed (benign neoplasm = 65.32%; malignant neoplasm = 80%). Within the multivariate regression model, children with benign neoplasms were 2 times more likely to undergo oophorectomy than those without a diagnosis for an ovarian lesion (OR = 2.03, *p* < 0.001). Children with a malignant neoplasm were almost 5 times more likely to receive an oophorectomy (OR = 4.82, *p* = 0.001).

**Table 1:** Oophorectomy rate by risk factor

Diagnosis	n	%	Logistic regression			Multivariable adjusted		
			OR	CI	p value	OR	CI	p value
No diagnosis	1.386/ 2.821	49.13	–	–	Ref	–	–	Ref
Ovarian cyst	1.314/2.597	50.60	1.06	0.95–1.17	0.28	1.09	0.97–1.22	0.12
Benign neoplasm	1.019/1.560	65.32	1.95	1.71–2.21	< 0.001*	2.03	1.78–2.33	<0.001*
Malignant neoplasm	24/30	80.00	4.14	1.68–10.16	0.002*	4.82	1.91–12.1	0.001*
Year								
2003	775/1.224	63.32	–	–	Ref	–	–	Ref
2006	742/1.241	59.79	0.86	0.73–1.01	0.07	0.90	0.76–1.07	0.24
2009	839/1.499	55.97	0.73	0.63–0.85	< 0.001*	0.75	0.64–0.89	0.001*
2012	675/1.303	51.80	0.62	0.53–0.73	< 0.001*	0.65	0.55–0.77	< 0.001*
2016	406/908	44.71	0.46	0.39–0.55	< 0.001*	0.49	0.41–0.59	< 0.001*
2019	306/833	36.73	0.33	0.28–0.40	< 0.001*	0.35	0.29–0.42	< 0.001*
Hospital type								
Rural	337/523	64.44	–	–	Ref	–	–	Ref
Urban, Non–teaching	1.168/1.959	59.62	0.81	0.66–0.99	0.046*	0.87	0.70–1.07	0.20
Urban, teaching	2.166/4.396	49.27	0.53	0.44–0.64	< 0.001*	0.64	0.53–0.79	< 0.001*
Income quartile, by zip code								
Lowest	1.084/1.897	57.14	–	–	Ref	–	–	Ref
25–50%	922/1.693	54.46	0.89	0.78–1.02	0.10	0.87	0.76–1.01	0.056
51–75%	882/1.672	52.75	0.83	0.73–0.95	0.009*	0.81	0.70–0.93	0.003*
Highest	799/1.629	49.05	0.72	0.63–0.82	< 0.001*	0.68	0.59–0.79	< 0.001*

*p-value less than or equal to a significance level of 0.05

Over time, rates of oophorectomy declined. In 2003 the rate of oophorectomy was 63.32% compared to 36.73% in 2019. Year of admission was a significant risk factor for oophorectomy within univariate and multivariate regression models. Children who presented in 2019 were 65% less likely to undergo oophorectomy compared to those in 2003 (OR: 0.35, *p* < 0.001). Hospital type was also a significant risk factor. Children in urban teaching hospitals were less likely to undergo oophorectomy than those in rural hospitals (OR: 0.64, *p* < 0.001).

Focusing on income, 57.14% of children within the lowest income quartile underwent oophorectomy versus 49.05% within the highest income quartile. Within the multivariate regression model, children within the highest income quartile had decreased odds of oophorectomy and were 32% less likely to receive this procedure (OR: 0.68, *p* < 0.001).

## Discussion

Overtime, the rate of oophorectomy amongst pediatric patients with ovarian torsion has decreased. This change may largely be attributed to new protocols and practitioner education recommending ovarian salvage. In a 2010 study utilizing the KID database from 2000, 2003 and 2006 it was reported that 58% of children diagnosed with ovarian torsion underwent oophorectomy. Moreover, the odds of having an oophorectomy decreased by 5% with each subsequent year [1]. The findings from our current study support this trend, as on average 53.4% of patients had oophorectomy when including KID data beyond 2006.

Although advances have been made, this study found that even children with benign neoplasms were more likely to undergo oophorectomy suggesting there is much room for improvement. Historically, clinical practice has erred towards oophorectomy given concern for malignancy in the setting of an enlarged ovary. The appearance of the ovary may change drastically in the setting of torsion making it difficult to determine on preoperative imaging if a tumor is present [12]. Although tumor markers have proven some utility in preoperative risk stratification of pediatric ovarian neoplasms, ovarian torsion is an acute surgical problem requiring immediate intervention [13]. In most cases of ovarian torsion, preoperative tumor markers are unavailable. Therefore, an argument can be made that there is surgeon to surgeon subjectivity regarding what appears to be benign at the time of surgery. An abundance of caution in the face of uncertain malignancy might push some surgeons towards oophorectomy. However, the reality is that malignancy is extremely uncommon in cases of ovarian torsion. Based upon a review of existing studies, Cass et al. estimated that approximately 2% of adult cases of ovarian torsion are secondary to malignancy [7]. Moreover, a study by Oltman et al. which focused on pediatric ovarian torsion reported a similar malignancy rate at 1.8% amongst a combined 707 cases reviewed between the literature and their study cohort [14]. A similar study by Lawrence et al. focused on children presenting with an ovarian mass and found that the risk of malignancy was lower amongst those with concurrent ovarian torsion versus those without (10 % v. 17%) [15]. Within our current study, the prevalence of malignancy was notably lower than in previous studies with a reported incidence of 0.43% (*n* = 30).

Given the low rates of pediatric ovarian malignancy in the setting of ovarian torsion, The American College of Obstetricians and Gynecologists recommends that regardless of the appearance of the ovary surgeons forego oophorectomy [16, 17]. Within the pediatric population, fertility preservation is an important consideration. A 10 year study by Geimanaite et al. found that 4–6 weeks after detorsion, almost 60% of patients had affected ovaries which returned to normal size. More importantly, all patients who had undergone detorsion retained folliculogenesis [18]. Even in the presence of a dusky appearing ovary, there was no significant relationship between ovary appearance and number of follicles on follow-up ultrasound [10]. A recent study found that patients who underwent detorsion secreted almost twice as much anti-Mullerian hormone (AMH) in comparison to those who underwent oophorectomy [19]. AMH is commonly used as a marker of ovarian reserve, thereby further highlighting the importance of ovarian preservation within this population. Future studies should continue to monitor trends in pediatric oophorectomy.

Another key finding of our study is that children from lower income quartiles and rural communities had increased rates of oophorectomy. Within this cohort we identified that children within urban teaching hospitals had higher rates of ovarian salvage than those in rural hospitals when adjusting for other risk factors (OR: 0.64, *p* < 0.001). Previous studies have also found that children from lower income were more likely to undergo oophorectomy [10]. A recent Texas-based study also found that children treated at non-teaching hospitals were almost twice as likely to undergo oophorectomy [20]. Similar results were found in another study which found that regardless of ongoing trends pushing for ovarian preservation, children at non-teaching hospitals were more likely to undergo this procedure [21].

Although there is no clear explanation for this pattern, one argument is that this disparity may be secondary to increased specialist availability at a teaching institution. At a teaching hospital, a less experienced surgeon may be more able to seek intraoperative advice from their senior and/or gynecologist colleagues. A Canadian study found that the presence of a pediatric gynecologist within the operating room significantly increased the possibility of ovarian preservation. Other protective factors identified within that study were early diagnostic imaging, preoperative ultrasound, and time to intervention. Ultimately, Hubner et al. were able to apply a quality improvement initiative which saw a remarkable increase in ovarian preservation rates from 47.6 to 85.6% across twenty-five years in a major children’s hospital. This continuous quality improvement (CQI) project entailed: continued medical education of emergency and primary care physicians, incorporation of a 6 week pediatric surgery curriculum into pediatric gynecology fellowship, and the creation of a standardized care pathway between the Gynecology, Oncology, and Pediatric Surgery departments [22]. Within the US, further research and quality improvement projects are warranted to explore the risk factors and possible preventative measures to increase rates of ovarian preservation in rural communities.

There are several notable limitations of this study. Firstly, available data within the KID’s database is contingent upon hospital participation. Thus, there is not only the potential to fail to capture cases of ovarian torsion from non-participating institutions, but this may also introduce bias within our analysis. Our study relies on post-discharge diagnosis codes within the KID’s database. As a result, it is possible that there are inappropriately coded diagnoses. It is possible that patients who were coded as having ‘no diagnosis’ were misclassified or potentially had a missed diagnosis. This potential error can impede data quality. Given the nature of the database we were also unable to determine the type of surgeon involved in management of ovarian torsion and their surgical approach. This presents two potential areas of cofounding. As aforementioned, gynecologists were less likely than pediatric surgeons to perform oophorectomy [11]. Therefore, if many gynecologists were included within our study cohort the reported rates of oophorectomy may be less representative of actual practice amongst pediatric surgeons. Regarding surgical approach (i.e. open v. laparoscopic) the procedure codes provided within this database provide no insight into this information. Hence, we are unable to identify cases of laparoscopic to open conversion. This is a notable limitation, as a recent study by Huerta et al. utilizing the National Readmissions Database (NRD) determined that oophorectomy was more common following an open versus laparoscopic approach (59% open). More importantly, our inability to discuss surgical approach limits our discussion regarding specific measures for possible quality improvement. Huerta et al. determined that both laparoscopic detorsion and ovarian preservation were underused—especially within non-teaching hospitals [23]. Inclusion of information regarding surgical approach may have added further nuance to this discussion. Undoubtedly, future studies focused on provider practice may provide insight to needed improvement initiatives.

There were further limitations within our data as we were unable to track the rate of bilateral ovarian masses. A recent single institution study focusing on ovarian torsion during the COVID epidemic identified that 4% of pediatric patients presented with bilateral ovarian torsion [24]. The presence of bilateral ovarian torsion may impact operative decisions and the likelihood of ovarian salvage. Currently, there is limited evidence to explore this possible association; thereby, presenting a possible future research opportunity.

Furthermore, although this study identified a significant difference in rates of ovarian salvage in rural versus urban environments, within the current database we were unable to factor in the impact of distance traveled on rates of oophorectomy. It is possible that children travelling longer distances to receive care may have longer duration of symptoms which may influence surgeon decisions regarding ovarian salvage. Future research may benefit in exploring this relationship as it may further explain the disparity observed within our analysis.

Despite these limitations, one key strength of this study is the generalizability of study findings as our analysis was completed within a robust database reflecting a diverse patient population.

## Conclusion

Within the US there has been a notable decline in rates of oophorectomy amongst pediatric patients presenting with ovarian torsion. This decline likely represents amended guidelines and improved practitioner education. Despite advances made in the last 20 years, rates of oophorectomy remain high in spite of the low rate of ovarian malignancy (0.43%). Children from lower income families and rural communities are more likely to undergo oophorectomy, further highlighting a need to adapt current surgical practice and education to combat this disparity.

## Data Availability

No datasets were generated or analysed during the current study.

## Abbreviations

KID: Kids’ Inpatient Database
AMH: Anti-Mullerian hormone
